# Intensive care for severe acute respiratory syndrome coronavirus 2 (SARS-CoV-2) in a makeshift ICU in Wuhan

**DOI:** 10.1186/s13054-020-02914-6

**Published:** 2020-05-06

**Authors:** Xiao Lu, Shanxiang Xu

**Affiliations:** Department of Emergency Medicine, Second Affiliated Hospital, Zhejiang University School of Medicine, No.88, Jiefang Road, Hangzhou, 310009 China

Dear Editor,

According to the control policy of SARS-CoV-2 in Wuhan, more than 20 new hospitals were built during February 2020 for the admission of all patients. Although there were enough ICU beds for almost all critically ill patients in Wuhan before the epidemic, the shortage of ICU beds in Wuhan was also a big problem we faced due to the large number of SARS-CoV-2 patients who required ICU care [1]. Many ICU doctors from emergency departments and other departments in different cities have come to Wuhan to help the patients.

The medical team from the Second Affiliated Hospital, Zhejiang University School of Medicine contains 120 nurses, and 42 doctors took over one makeshift ICU that was transformed from an orthopedics ward just before we arrived in Wuhan on February 14, 2020. The patients in this ward had been transferred to hotels or their homes, and medical resources were prepared before new patients were admitted. Due to the short supply of medical resources in Wuhan, we transferred most of the resources and protective equipment needed in this ICU from our hospital, e.g., mechanical ventilators (MV), high flow nasal cannula (HFNC) devices, noninvasive ventilators (NIV), monitors, ultrasound machines, and ECMO.

There were 40 beds in this temporary ICU but no negative pressure room. Two sterilization robots were used to keep the air clean, and we also set up a few airborne disinfection systems in the units and offices. As the supply of oxygen was restricted, only six MVs were allowed to be used at the same time, which meant only a few patients could be simultaneously treated with the MVs. One ECMO was removed from our hospital but not used in our ICU because all patients who needed mechanical ventilation treatment were over 80 years old and their families declined ECMO treatment. It is recommended that a full evaluation be performed on patients before ECMO is performed in any nonstandard ICU, as there will be more chances for complications during the treatment and there is a huge workload placed on nurses. The high-flow nasal cannula and noninvasive ventilators were more commonly used in our ICU. The running process of the makeshift ICU is shown in Fig. 1. Many patients were transferred from the mobile hospitals such as LeiShenShan and HuoShenShan hospitals, hotels, or their homes when their condition became serious and required critical care. Sixty-one patients were admitted in the 10-day period after we arrived, and 11 of them had severe ARDS requiring MV treatment. Five patients died during the treatment, four patients died of SARS-CoV-2 despite treatment with MV, and one died from dilated cardiomyopathy while awaiting a heart transplant.

**Fig. 1:**
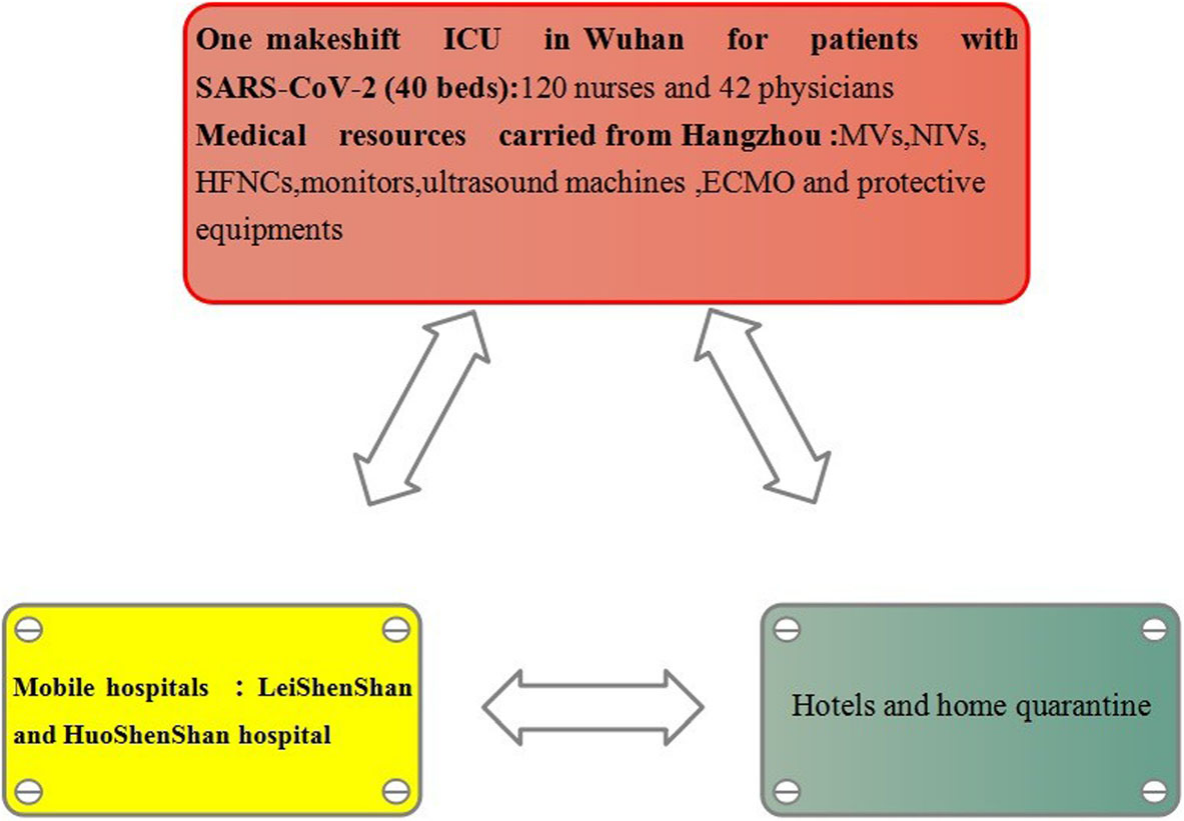
The running process of one makeshift ICU in Wuhan

Protecting the workforce was another critical challenge. Most of the doctors had no experience working in such a makeshift ICU, which made them nervous and uncomfortable [2]. The doctors were divided into eight groups of 4–5 doctors each (running 6-h shifts), so they could have more time to rest and relax. One psychiatrist in our team helped us to resolve psychological problems such as insomnia and anxiety. It was very difficult for doctors and nurses to manage so many critical patients in this ICU. We must remain vigilant to avoid further infection, as 2050 doctors have been infected in the hospital since the epidemic first began. As we know, there have been over 10 makeshift ICUs like this in Wuhan that have saved the lives of more than 1000 critically ill patients. We believe the doctors from different cities in China will continue to do outstanding work in such a harsh environment.

## Data Availability

Not applicable.
